# Dr. Austin Flint, and His Address in Medicine

**Published:** 1886-06

**Authors:** 


DANIEL'S

### Medical ★ Journal,

PUBLISHED MONTHLY AT

AUSTIN, TEXAS.

F. E. DANIEL, M. D., Editor and Publisher

Subscription Price: Two Dollars per Annum in Advance.

Papers for publication in this Journal should be in the hands of the Editor by the 10th of the month preceding the month in which it is wished to appear; they must be original,—never having appeared in print elsewhere, and must be contributed excluisvely to this Journal.

Contributions to the department of Original Articles are cordially invited from the professiom of Texas, expecially. What is most desired is Clinical reports of cases, essays and short practical articles on any medical topic; also solicited reports of proceedings of societies, clubs, etc., and items of medical news of general interest to the profession, such as notices of appointments of physicians, removals, changes, marriages, deaths, etc.

### EDITORIAL.

### AUSTIN FLINT, AND HIS ADDRESS IN MEDICINE.

We have received from the Publishers, the Messrs. Appleton, 1, 3 and 5 Bond Str. N. Y., a little book entitled, "Medicine of the Future," it being the address prepared by Austin Flint, (Senior), M. D. L. L. D., and which was to have been delivered by him before the British Medical Association this year. It is printed in clear large type on Bristol card paper, and bound in fine cloth and boards, with gilt title and edge. It is illustrated by an exquisitely artistic steel engraving of the author, which is, moreover, an excellent likeness. The book seems to have been gotten up as a *souvenir*, for presentation, and has some ten or a dozen blank leaves in front and back; suitable for inscriptions.

These pure white surfaces may well typify the spotless character of the life it is sought to commemorate; but there were no *blank leaves* at either end of his useful and eventful life; for beginning early, he worked constantly, and ceased not till the very day of his death. He literally died in the cause to which he had given the whole of his busy life,—this exquisite essay being one of the last, if not the last production of his fruitful brain and pen. Every physician who loved the *Great Father of American Medicine*, the MODERN HIPPOCRATES, (and who did not; who with soul so dead as not to reverence his memory?) should have a copy of his "Address" as a keepsake.

Austin Flint was no ordinary man;—

"Cast in the massive mould
Of those high statured ages old
Which into grander forms our mortal metal ran,"

he stood,—alone in his grandeur in the western world! "Like some tall cliff that lifts its mighty form," he towered above his fellows,—the peerless physician—author—man;—or like that matchless orb which majestically rolls through the ethereal realms of the upper deep—the monarch of the midnight skies,—whose ray of transcendent brilliancy distinguishes it in the star-set heavens, he shone in the firmament of the professional world, the Jupiter of Modern Medicine! He too, was surrounded by *Satelites*; and of a brilliant constellation, was the grand central,—the supreme figure! He will never be forgotten! He lives and breathes in his works yet fresh and warm from his mighty brain; and so long as the human heart shall beat, and the human lungs give out the "respiratory murmur,"—so long will Austin Flint's name be associated with their sounds. The taps on his little pleximeter, so familiar in the wards of old Bellvue will echo down the corridors of time, till time shall be no more!—and as with ceaseless course the circling centuries roll away, Austin Flint will be canonized. He will live for aye in the annals of medicine, and in the hearts of its devotees; even as the Sage of Cos lives to-day; and future generations will esteem him, as we, of the present, do his prototype—less a man than a God!

